# Strain-Controlled Fatigue Behavior and Microevolution of 316L Stainless Steel under Cyclic Shear Path

**DOI:** 10.3390/ma15155362

**Published:** 2022-08-04

**Authors:** Xinna Liu, Shuai Zhang, Yanmei Bao, Zhongran Zhang, Zhenming Yue

**Affiliations:** 1 Rongcheng Campus, Harbin University of Science and Technology, Rongcheng 264300, China; 2 School of Mechanical, Electrical and Information Engineering, Shandong University at Weihai, Weihai 264209, China; 3 Naval Architecture and Port Engineering College, Shandong Jiaotong University, Weihai 264310, China

**Keywords:** cyclic shear, strain amplitude, cyclic response, martensitic transformation

## Abstract

Based on the twin bridge shear specimen, the cyclic shear experiments were performed on 1.2 mm thin plates of 316L metastable austenitic stainless steel with different strain amplitudes from 1 to 5% at ambient temperature. The fatigue behavior of 316L stainless steel under the cyclic shear path was studied, and the microscopic evolution of the material was analyzed. The results show that the cyclic stress response of 316L stainless steel exhibited cyclic hardening, saturation and cyclic softening, and the fatigue life is negatively correlated with the strain amplitude. The microstructure was analyzed by using electron back-scattered diffraction (EBSD). It was found that grain refinement and martensitic transformation during the deformation process led to rapid crack expansion and reduced the fatigue life of 316L.

## 1. Introduction

With excellent strength, corrosion resistance, heat resistance, ductility and machinability, 316L stainless steel has been widely used in industrial applications [1,2,3]. The steel is usually made into parts that are subjected to certain cyclic loads at low, ambient or high temperatures, which will lead to fatigue failure of the parts [4]. Therefore, in order to improve the service life of materials under cyclic loading conditions, many scholars have continued to conduct in-depth studies on the microstructure and deformation mechanism of materials under cyclic loading. Pham et al. [5] conducted axial fatigue loading experiments on AISI 316L at room temperature, and found that the cyclic deformation response can be divided into three stages: cyclic hardening, followed by cyclic softening, and, finally, cyclic saturation before failure. In total, 316L stainless steel exhibits a cyclic hardening softening response in strain-controlled cyclic torsion [6]. The cyclic stress behavior of 316LN is determined mostly by the internal stress under non-proportional loading, and the initial cyclic hardening is greatly enhanced by the increase in loading non-proportionality [7]. Facheris et al. [8] carried out a set of uniaxial, torsional and multiaxial low-cycle fatigue and strain-controlled ratcheting tests. The effect of ratcheting on the mechanical response was found to be quantitatively stronger, causing a more pronounced drop of fatigue life. Zhou et al. [9] developed a cyclic constitutive model implemented to describe the cyclic behaviour of 316L including the hardening/softening and the strain range memory effect. During the uniaxial ratchetting deformation of 316L stainless steel, the dislocation density increases progressively, and twinning and strain-induced martensitic phases do not occur within the specified number of cycles [10]. Observations of the life-terminated dislocation arrangements by transmission electron microscopy showed that the dislocation microstructure depends essentially on the plastic strain amplitude, which in turn is strongly correlated to the stress amplitude and average stress [11]. It is believed that the austenite phase in some stainless steels is in a metastable state at ambient temperature due to the amount of Cr and Ni. In the process of monotonic or cyclic deformation, the deformation of the metastable austenite leads to the phase transformation from initial fcc austenite (γ) to final stable martensite(α′) [12,13,14,15]. Li et al. [16] performed a series of cyclic tests on 304L stainless steel with different loading paths, which showed that the growth rate of martensite content became higher and its distribution in the austenitic matrix was more uniform as the loading path increased non-proportionally. The sensitivity of the martensite induced by deformation depends on the chemical composition, temperature, degree of plastic deformation, and strain rate [17,18]. At the same time, strain-induced martensitic phase transformation can affect the ductility of austenitic stainless steels. It is believed that austenitic stainless steels exhibit significant work hardening, leading to the transformation from austenite to martensite [19]. Okayasu et al. [20] conducted an in-depth study of monotonic loading of austenitic steels at room and low temperatures and modeled the formation of martensite due to deformation. Das et al. [21] studied the effect of strain rate on strain induced martensite transformation characteristics. Some scholars also predicted the life of materials under fatigue loading. Branco et al. [22] developed the total strain energy density method to evaluate the fatigue life of notched specimens subjected to multiaxial loads, and the fatigue master curve can be efficiently generated from only two uniaxial strain-controlled tests, and a set of numerical simulations performed via single-element elastic-plastic models. Pelegatti et al. [23] established a robust procedure for durability design to estimate the strain–life curve, which was an effective way to deal with durability problems. Li et al. [24] proposed an energy-based model to predict the creep-fatigue, combined with high-low cyclic loading life. Because the fatigue fracture surface morphology can provide additional information for the analysis, many people have made a detailed analysis of the fracture surface. Fatigue fracture morphology is studied at different scales, and the most common fracture studies are observed using scanning electron microscopy (SEM) [25,26]. Post-failure applications of fracture surface measurements help to study the fatigue life and the fatigue damage accumulation in notched specimens subjected to severe stress gradient effects [27]. Macek et al. [28] studied the effect of asynchronous axial torsional strain-controlled on the fracture surface behavior of thin-walled tubular austenitic steel specimens. They also found that features of the post-failure fractures were related to the loading conditions and the fatigue life and the loading path significantly affects the surface topography. One of their conclusions is that the total volume fraction of martensite decreases when the sample temperature and the strain rate are increased. However, to date, although most researchers have studied the effects of stretching or combined torsion and tension on the microstructure and fracture behavior of austenitic stainless steel, few studies have studied the fatigue behavior of austenitic stainless steels under cyclic shear path.

Various forms of shear specimens have been used in the field of materials research, mainly applied to analyze forming properties and fracture behavior. Although the use of shear specimen’s own geometry in the universal testing machine can achieve shear loading, it will produce a reaction torque in the process of loading, making the specimen deformation, resulting in shear path change [29,30]. Yin et al. [31] proposed the specimen for in-plane torsional experiments, which used the thinning method of machining grooves to form the experimental area. The shear loading is then completed by the relative rotation of the inner and outer rings. However, due to the need for mechanical machining of the specimen, there must be a hardening layer of interference, and the uniformity of the machined thickness cannot be guaranteed during the machining of the thin plate. Brosius et al. [32] proposed a new twin bridge shear specimen which can achieve large shear deformation by shearing the middle experimental region through the relative rotation of the inner and outer rings, and the stress-train change is measured by the torque sensor and the rotation angle. The experimental process does not have the high response movement of the unilateral shear specimen, no compensation is required, and the two experimental regions of the twin bridge shear specimen have the same deformation direction, which is suitable for studying the plate anisotropy.

Therefore, in this study, the principle of the twin bridge shear experiment is chosen to optimize the design of a shear fatigue test. Extensive strain-controlled experiments were conducted on 316L austenitic stainless steel by means of a designed cyclic shear fatigue testing machine. The main purpose is to investigate how deformation-induced martensite affects cyclic deformation and fatigue life, and to evaluate the effect of cyclic shear on the microstructural evolution of 316L austenitic stainless steel by microscopic analysis of test samples.

## 2. Experimental Procedure

### 2.1. Experimental Setup and Specimens

**Figure 1:**
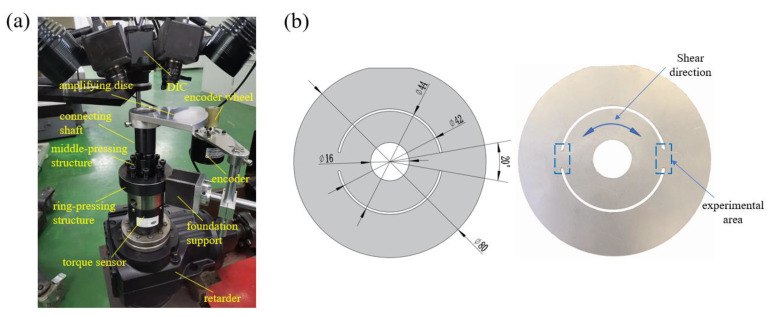
(**a**) Cyclic shear fatigue testing machine, (**b**) twin bridge shear specimen.

To study the fatigue behavior of materials under the cyclic shear path, a cyclic shear fatigue testing machine based on the twin bridge shear specimen was designed. The physical drawing of the machine and the geometry of the test piece is shown in Figure 1. The reducer in the base provides power to the power shaft so that the power shaft can rotate. The torque sensor is fixedly connected with the reducer housing, so that the Torque sensor is relatively static with the base. The outer and inner rings of the specimen are in contact with the upper surface of the torque sensor and the upper surface of the power shaft, respectively. The middle pressing structure passes through the circular hole of the middle clamping plate and the central hole of the test piece in turn, and is connected with the power shaft through threads. The inner ring of the specimen is firmly fixed to the power shaft by rotating the bolt on the middle pressing structure. When the power shaft rotates, the inner ring of the specimen rotates with it. The ring-pressing structure is fixedly connected with the torque sensor by threads. The outer ring of the specimen is firmly fixed to the torque sensor by rotating the bolt on the ring-pressing structure. Therefore, when the reducer rotates, the power shaft and the inner ring of the specimen rotate together, but the outer ring of the specimen remains relatively stationary with the equipment, thus making the inner and outer rings of the specimen rotate relatively, and then completing the shear loading of the specimen. The connecting shaft is fixedly connected with the middle pressing structure through threads. The power shaft drives the connecting shaft to rotate, thus ensuring that the connecting shaft will not be deformed by the torsional force during the loading process. The amplifying disc is fixedly connected with the connecting shaft and rotates together with it, which can further improve the resolution of angle detection. One end of the encoder is fixed on the ring-pressing structure by magnetic suction, and the other end is in contact with the amplifying disc, so that the encoder can be driven to rotate when the amplifying disc rotates, thus recording the rotation angle. The specimen fixation method adopted by this device will not cause large deformation of the positioning piece due to the influence of external force, nor will it cause deformation of the key structure due to the action of torque, to better ensure the consistency of the experimental results [33]. At the same time, because one end of the encoder is fixed to the ring-pressing structure, and the other end is in contact with the amplification disc, this connection method effectively shields the impact of the transmission gap of the system on the experimental accuracy, so that the angular resolution can reach 0.002°.

### 2.2. Experimental Materials and Methods

**Figure 2:**
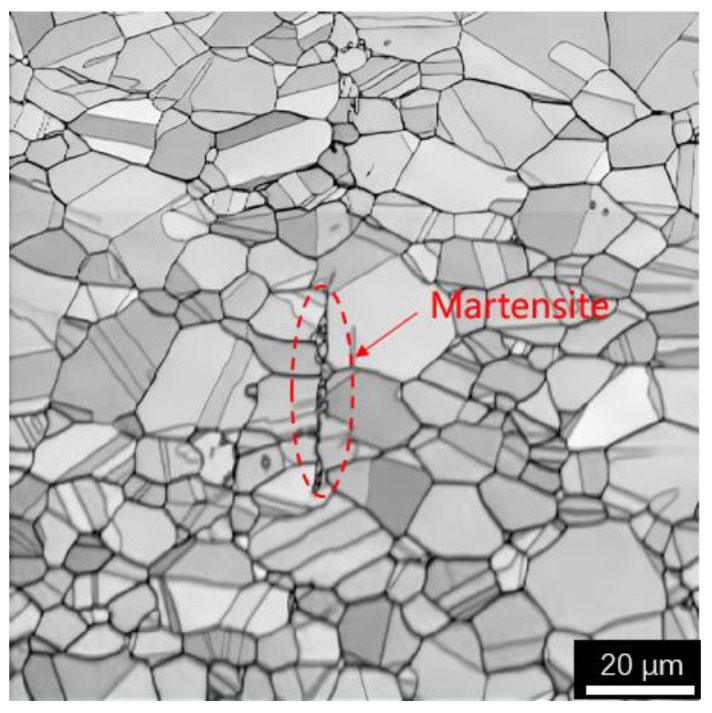
Microstructure of 316L before fatigue experiments.

The commercial 316L metastable austenitic stainless steel sheet produced by the hot rolling process in this study has excellent heat resistance, ductility, corrosion resistance, and mechanical properties. The specimen used for the fatigue test is shown in Figure 1b. Figure 2 shows the metallographic image of the material with 98% austenite content. The chemical composition of the specimen measured by using EBSD is shown in Table 1.

**Figure 3:**
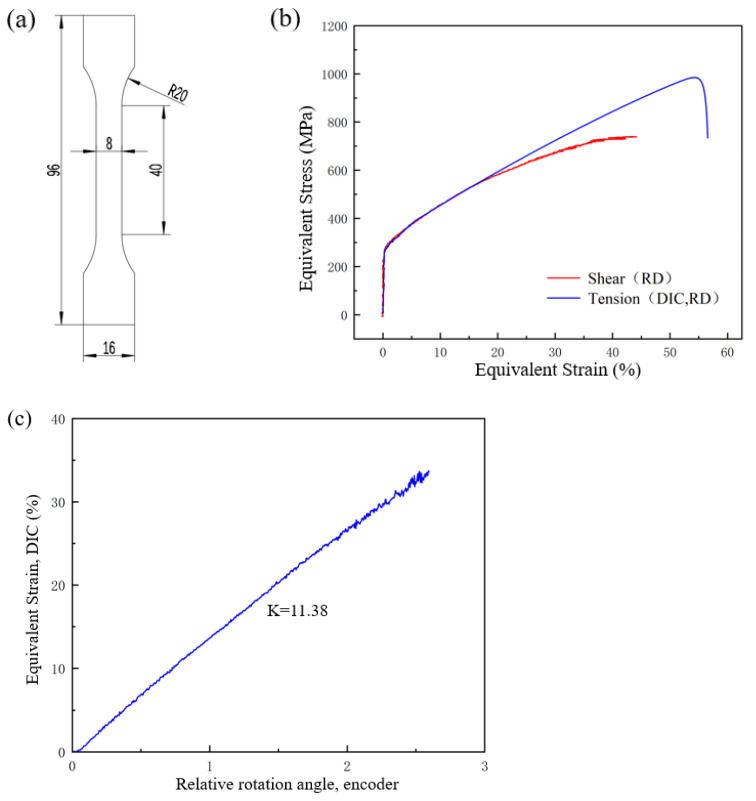
(**a**) Specimen geometry in monotonic tensile test, (**b**) comparison of tensile and shear, (**c**) relationship between equivalent strain and rotation angle.

In order to verify the relationship between the rotation angle of the inner and outer rings and the strain, the monotonic shear loading experiment of 316L was carried out by using DIC. The angle acquisition of DIC and the encoder of the testing machine adopt the same time interval and start at the same time. The relationship between strain and angle is determined by interpolation [34]. The geometry of the 316L tensile specimen is shown in Figure 3a. The equivalent shear stress–strain and tensile stress–strain curves of 316L is shown in Figure 3b. It can be seen from the figure that the yield point and strengthening process of 316L are very similar under the tensile and shear paths, and stretching has better elongation. At the stage of failure, the curve decreases rapidly under the tensile state and slowly under the shear state. The von Mises stress and strain studied in this paper were regarded as equivalent stress and strain, and the von Mises equation was used to calculate the equivalent stress and strain.

From Figure 3c, a linear relationship between the rotation angle and the equivalent strain can be observed, which is consistent with the research conclusion of Yin et al. [34]. Therefore, the results of Figure 3c can be used to determine the relationship between the magnitude of strain amplitude and the loading angle in subsequent studies. At room temperature, the strain rate (dε/dt) was constant at 2 × 10^−2^ S^−1^ and the strain-controlled symmetric shear loading tests were carried out under different total strain amplitudes (△ε/2) ranging from 1 to 5%, the strain ratio was −1 (R = ε_max_/ε_min_ = −1). Three tests were carried out under each strain condition, and a total of 15 groups of tests were carried out. After the fatigue test, the test section in the specimen was cut off by wire cutting. Then specimens were ground and polished with sandpaper of different specifications, and polished with 0.5 μm diamond polishing fluid and polishing cloth until the surface of the specimen had no scratches. Finally, these specimens were electropolished at room temperature. EBSD was used to analyze the original specimens and cracked specimen. The EBSD data were processed by ATEX software without data cleaning to obtain microstructure in different states.

## 3. Experimental Results and Analysis

**Figure 4:**
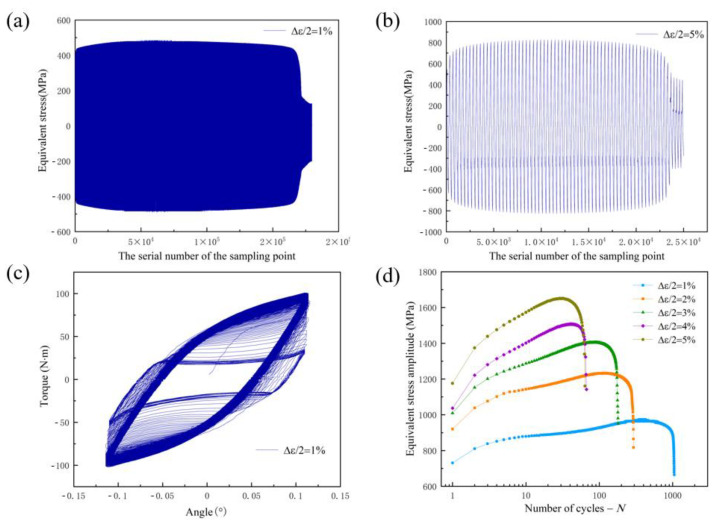
(**a**) Stress changes during 1% strain amplitude loading, (**b**) stress changes during 5% strain amplitude loading, (**c**) curves of relative rotation angle of specimen and torque under 1% strain amplitude, (**d**) cyclic stress response curves with different strain amplitudes.

Figure 4a,b show the stress response at strain amplitudes of 1% and 5%. The initial loading direction was defined as the positive direction of stress and strain. It can be seen from the figure that the distribution of the maximum positive stress and negative stress is approximately symmetrical under the same strain amplitude. The stress increases obviously at the initial stage of loading. With the increase in the number of cycles, the material was continuously hardened, then continuously softened until failure. Figure 4c shows the variation curves between angle and torque when the strain amplitude was 1%. The changes in shape are caused by the continuous hardening of the material, followed by continuous softening until failure. With the increase in the number of cycles, the strength of the material gradually increases, making the hysteretic curve gradually steeper. When the material hardens to a certain extent, the strength of the material begins to decrease gradually, and the hysteretic curve flattens gradually. The relationship between the stress amplitude and the number of cycles N under five different strain amplitudes is plotted in Figure 4d. It can be seen from the figure that the material hardens rapidly at the beginning of loading and the hardening amplitude increases with the increase in the strain amplitude, that is, the cyclic hardening of 316L is affected by the loading amplitude. With the increase in the strain amplitude, the stress amplitude increases, and the fatigue life decreases.

**Figure 5:**
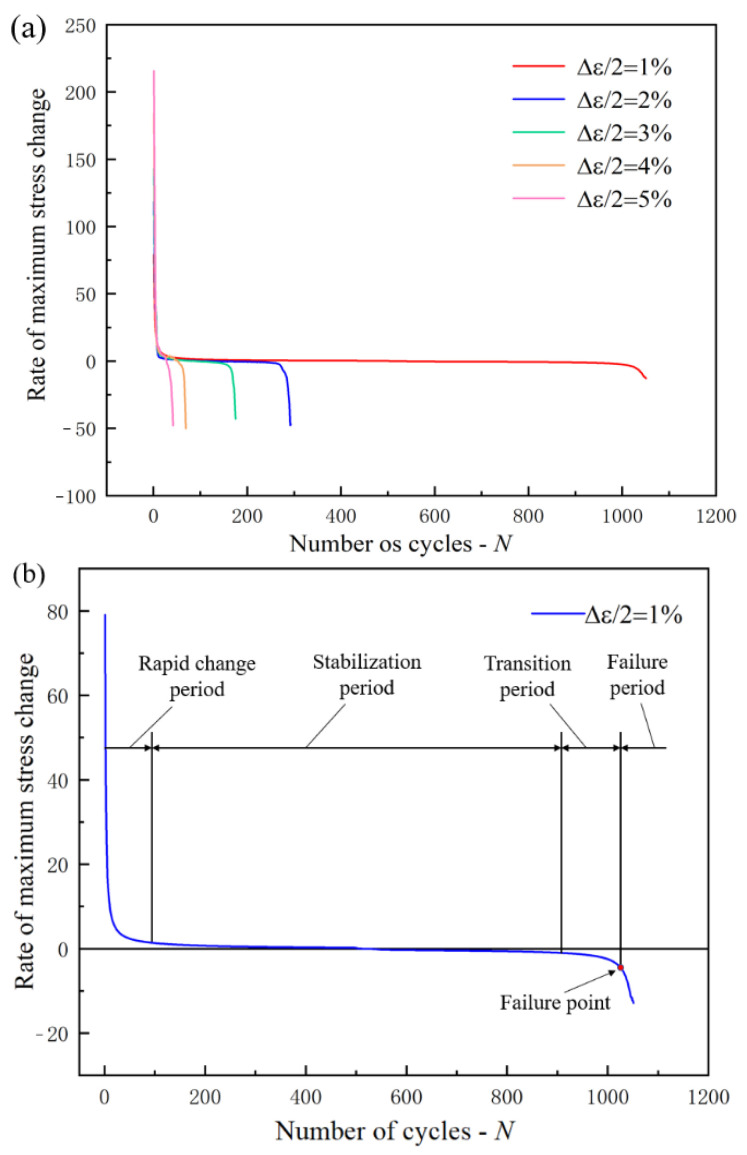
The change rate of the cyclic stress response curve of fatigue life: (**a**) the change rate of stress response curve under different strain amplitudes, (**b**) the change rate of cyclic stress response curves during 1.0% strain amplitude.

The change rate of the cyclic stress response curve was used to analyze the stress change rate. The fatigue life of the specimen was defined as the number of cycles, and the first derivative of the stress amplitude in the fatigue life was carried out. The relationship between the stress change rate and the number of cycles under different strain amplitudes is shown in Figure 5a. As shown in Figure 5b, the fatigue life is divided into four stages by using 1% strain amplitude as an example: rapid change period, stabilization period, transition period, and failure period. Half the number of cycles at this failure point is defined as the half-life. In the rapid change period, the stress change rate decreases rapidly and tends to be stable gradually. The stabilization period includes the cyclic hardening and softening of the material. The hardening stage is above the zero axis and the softening stage is below the zero axis as shown in Figure 5b. With the increase in the number of cycles, the stress change in the hardening stage decreases linearly and the stress change rate in the softening stage increases linearly. The proportion of the stabilization period in the whole life cycle decreases with the increase in the strain amplitude. The stabilization period accounts for 76.5% of the whole life cycle during 1% strain amplitude. In other words, the length of the stabilization period determines the length of fatigue life. The longer the proportion of stabilization period, the longer the fatigue life. During the transition period, the stress change rate increases rapidly. At this time, obvious cracks have appeared in the specimen and the crack growth rate continues to increase. Finally, the material reaches the failure point, the specimen enters the failure period, and the stress change rate decreases rapidly, which indicate the fracture of the specimen.

**Figure 6:**
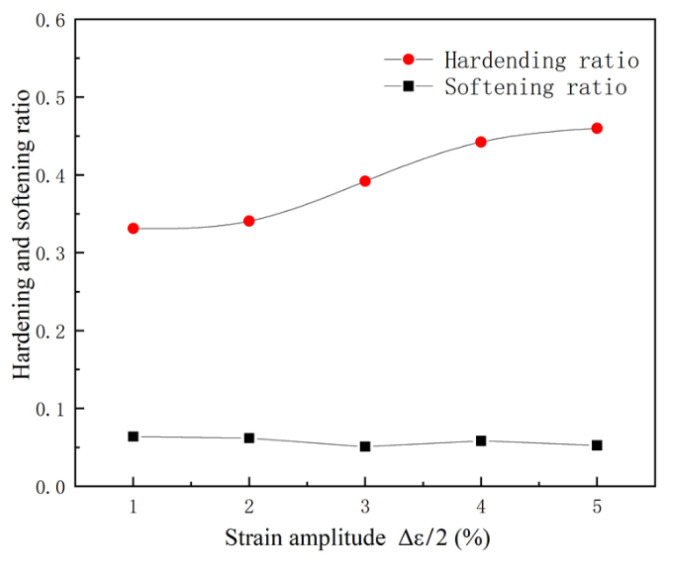
Cyclic hardening and softening ration under different strain amplitudes.

To further study the cyclic hardening and softening behavior of materials under different strain amplitudes, the cyclic hardening ratio (HR) and cyclic softening ratio (SR) are used to describe this phenomenon [33,35]. Figure 6 shows the relationship between the strain amplitude and cyclic hardening and softening. Under different strain amplitudes, the hardening ratio of the material increases with the increase in the strain amplitude and the softening ratio of the material almost remains unchanged. It is shown that the softening ability of 316L has little relation to the strain amplitude, and the strain amplitude affects the hardening ability of 316L.

**Figure 7:**
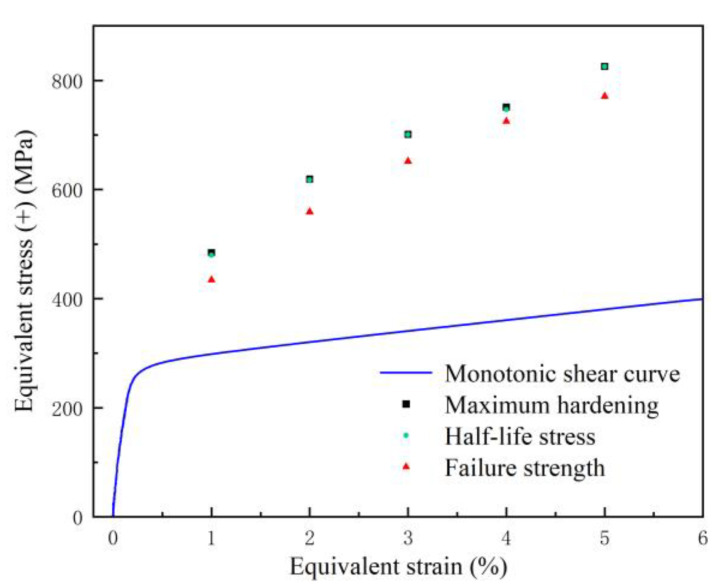
Monotonic shear curve, maximum hardening, half-stress, and failure strength relationship for 316L under cyclic shear path.

**Figure 8:**
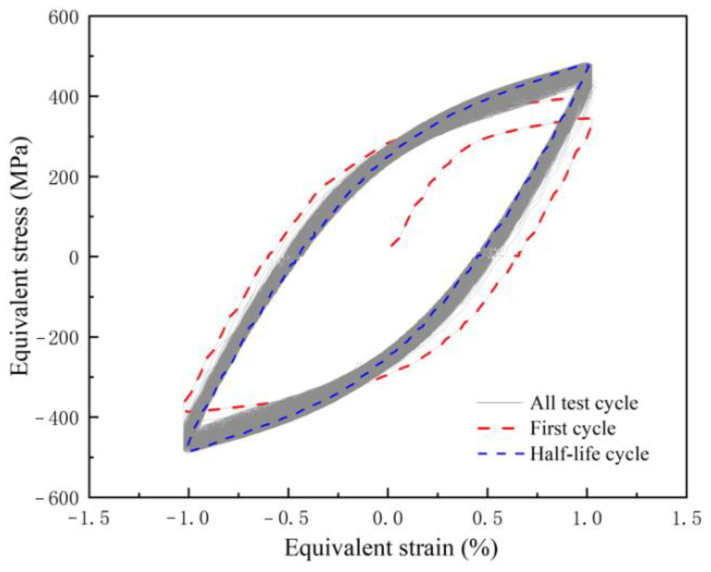
Hysteresis curve at 1% strain amplitude.

**Figure 9:**
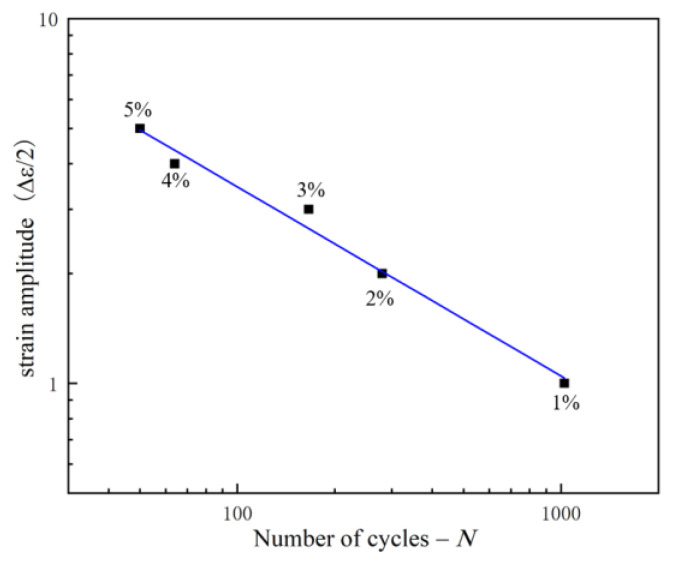
Fatigue life at different strain amplitude.

From the previous cyclic fatigue stress change diagram, it can be seen that the distribution of positive stress and negative stress is approximately symmetrical. In order to compare the stress change under cyclic fatigue with those under monotonic shear, the positive stress under cyclic fatigue was selected for analysis. The maximum positive stress at each strain amplitude was connected, which was defined as the maximum hardening curve (MH). The positive stress at the failure point at each strain amplitude was connected, which was defined as the failure strength curve (FS). According to the number of cycles at the failure point, the number of cycles corresponding to the half-life was calculated. Connecting the stress corresponding to the half-life of different strain amplitudes defined as the half-life stress curve (HS), MS, MH, HS, and FS were plotted in Figure 7. It can be seen from the figure that MH, HS, and FS are all approximately linearly related to the strain amplitude, and MH, HS, and FS are all higher than MS, which is caused by the hardening of the material under cyclic shear loading. MH and HS basically coincide at the same strain amplitude, which indicates that the hardening degree of 316L reaches the maximum at the half-life. To prove this point, the hysteresis curve at 1% strain amplitude is used as an example, as shown in Figure 8. It can be seen that the stress–strain curve at half-life is located at the maximum stress position of the entire hysteresis curve, which is the time of maximum hardening. In other words, the hardening and softening of 316L account for half of the fatigue life, respectively. Therefore, the strain amplitude under cyclic shear fatigue has a very important effect on the hardening and fatigue life of 316L. The fatigue life is approximately linear with the strain amplitude, and decreases linearly with the increase in the strain amplitude, as shown in Figure 9.

## 4. Microstructure Evolution during Cyclic Shear Loading

**Figure 10:**
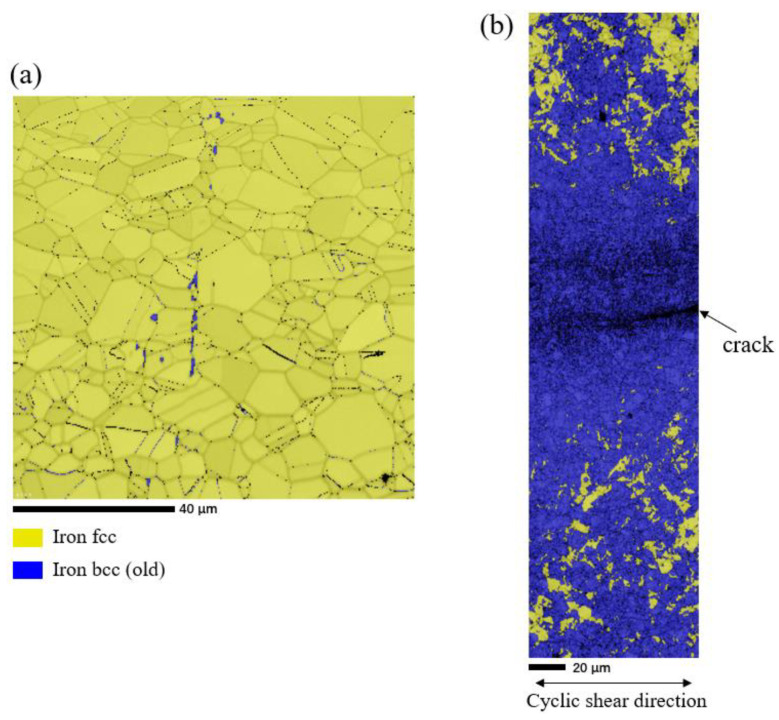
EBSD phase image of specimen fatigued cycle. (**a**) Phase image of the original specimen, (**b**) phase image of the specimen after experiment.

The EBSD technique was used to analyze the metallographic composition of the test specimens under cyclic fatigue shear path, and Figure 10 shows that the original material of 316L austenitic stainless steel was mainly austenite. The specimen produced a crack under the shear path, which was surrounded by martensite and the grain was refined. This means that the strain-induced austenite transforms into martensite with the crack initiation, and results in grain refinement around the crack. The tendency to the strain-induced martensite transformation has been explained in terms of the variation in the chemical free-energy difference between the austenite and martensite phases, referred to as the chemical driving force [18]. The hardening of austenitic stainless steels caused during fatigue cycle under a push-pull path is mainly due to deformation-induced martensite and dynamic strain aging (DAS). At room temperature, the decrease in fatigue life is due to the rapid crack growth induced by deformation-induced martensite hardening. The amount of martensite increases with the increase in strain amplitude, which is responsible for the rapid secondary hardening. Because no martensite was found at high temperature, the hardening was caused by dynamic strain aging [36]. Therefore, the hardening of 316L under cyclic shear path at room temperature is caused by martensite transformation. With the increase in cycle times, the number of martensite increases, which makes the crack propagate rapidly and leads to the decrease in fatigue life.

## 5. Conclusions

The strain-controlled fatigue properties of 316L austenitic stainless steel was investigated and the cyclic deformation microstructure was analyzed by EBSD in this work. The conclusions are as follows:

1. Under the cyclic shear path at room temperature, 316L exhibits cyclic hardening, saturation, and cyclic softening. The hardening rate is positively correlated with the strain amplitude.
2. The cyclic hardening and cyclic softening of 316L each account for approximately 50% of the life cycle, and the half-life coincides with the saturation period of cyclic hardening.
3. The deformation-induced martensitic transformation causes cyclic hardening of 316L austenitic stainless steel, whereas the fatigue life is reduced due to the rapid crack expansion caused by the deformation-induced martensitic transformation. The fatigue life is negatively correlated with the strain amplitude.

In conclusion, this study provided an in-depth investigation of the failure mechanism of 316L under shear cyclic path through experiments and microstructure observation, which provided a pre-theoretical guidance for the development of a life prediction model under cyclic shear path. The failure scenarios at different strain ratios and the development of life prediction models will be carried out in future work.

## Figures and Tables

**Table 1:** Chemical composition in weight percentage of the AISI 316L hot rolled plate.

C	Si	Mn	P	S	Cr	Ni	Mo	N
0.02	0.5	1.18	0.03	0.001	16.91	10.26	2.11	0.04

## Data Availability

Not applicable.
